# Flower, seed, and fruit development in three Tunisian species of *Polygonum*: Implications for their taxonomy and evolution of distyly in Polygonaceae

**DOI:** 10.1371/journal.pone.0227099

**Published:** 2020-01-10

**Authors:** Maher Mahmoudi, Fayçal Boughalleb, Giuseppe Pellegrino, Raoudha Abdellaoui, Nizar Nasri

**Affiliations:** 1 Université de Tunis El-Manar, Faculté des Sciences de Tunis, Tunis, Tunisia; 2 Laboratoire des écosystèmes pastoraux et valorisation des plantes spontanées et des microorganismes associés, Institut des Régions Arides, Médenine, Tunisia; 3 Department of Biology, Ecology and Hearth Sciences, University of Calabria, Rende (CS), Italy; Wuhan Botanical Garden, CHINA

## Abstract

*Polygonum* is the largest genus of *Polygonaceae* and 5 species are reported in Tunisia. In order to characterized flower, seed, and fruit development in *Polygonum*, flower and fruit of *Polygonium equisetiforme* (var. *graecum* and p*eyerinhoffi*), *P*. *aviculare* and *P*. *maritimum*, collected from Tunisia, were examined. Flowers are composed of five oblong tepals. *P*. *equisetiforme* and *P*. *aviculare* have whitish-pink distylous flowers with dimorphism of style, filament and anther height, pollen diameter and stigma size. In contrast, *P*. *maritimum* shows white homostylous flowers. The floral vasculature showed that the tepals are inserted in one whorl and their traces arise independently in 3+2 manner. The eight stamens are arranged in a 5+3 manner and the staminal bundles arise independently in the two whorls. The epidermis and endothecium cells width were higher in *P*. *maritimum* and the lowest endothecium width was observed in *P*. *aviculare*. *Polygonum aviculare* and *P*. *equisetiforme* showed circular pollen with shallow colpi and trilobite pollen shape with deep colpi, while *P*. *maritimum* rarely showed shallow colpi. The ovule is anatropous with basal placentation in *P*. *equisetiforme* and *P*. *aviculare* and apical placentation in *P*. *maritimum*. The young seed coat was formed by an endotesta with thick-walled cells, a mesotesta and exotesta with thin-walled cells and a tegmen composed of radially elongated cells. The fruits of the studied species are trigonous with ovate-lanceolate shape. In *P*. *aviculare*, the exocarp is thicker compared to the two other species, in *P*. *equisetiforme*, the mature exocarp consists of smaller rectangular cells with narrow cavities, and in *P*. *maritimum showed* a thinner exocarpIn conclusion, *P*. *equisetiforme* and *P*. *aviculare* are a typically distylous species from the morphological point of view and we discussed the significance of heterostyly in Polygonaceae. From this first morpho-anatomical study of *Polygonum* species in North Africa, we can conclude mainly that there is no significant difference between *P*. *equisetiforme* var. *graecum* and var. p*eyerinhoffi* supporting a taxonomic grouping of these two varieties.

## Introduction

The *Polygonaceae* are a large and cosmopolitan family of herbs, shrubs, climbers or trees which comprise approximately 30 to 49 genera and about 750 species [1, 2], geographically distributed widely in tropical, subtropical, and temperate regions [3]. *Polygonum* L. is the largest genus of *Polygonaceae* and comprises approximately 150 species in the world [4] distributed mostly in Europe, North Africa, and Western Asia. They are annual and perennial herbs, subshrubs or shrubs with woody stocks [5]. *Polygonum* species are, generally, characterized by having ochreae on nodes, prostrate or erect stems, alternate leaves, axillary flowers, tepals with dendritically branching main vein, eight to less stamens with a dilated base arranged in two whorls (inner and outer), and swollen filaments at the base with no visible nectaries [6, 7]. In Tunisia, the *Polygonaceae* are represented by 4 genera and 9 species [8].

The use of various characters, such as vegetative anatomy [9, 10], epidermal characters [10, 11] and pollen morphology [12, 13, 14] are of great importance. Moreover, the foliar morphological and anatomical investigation has effectively contributed in recognizing several segregates in the genus *Polygonum* L. Due to its stable character sets, the morphological traits of achene as well as seeds have become an important issue in species classification, providing a relevant taxonomically analysis at different hierarchical level [2, 15]. Information on achene anatomy of some *Polygonaceae* was presented by [16, 17]. It has now been suggested that the anatomy and morphology of *Polygoneae* achene are useful in systematic studies and implications [18].

From the beginning of the twentieth century, the floral structure and anatomy attracted the attention of several researchers such as [19, 20]. [21] studied the position and morphology of the floral nectaries of *Polygonum* and related genera.

The *Polygonaceae* are characterized by heterostyly as first described by [22]. The identification traits of *Polygonum* taxa are especially based on homo- or heterophylly, ochrea texture and shape and flower color [6]. Style-stamen dimorphism in *Polygonum* was well documented [23]. The genus *Polygonum* is found to be heterostylous, indeed *Polygonum chinense* (recently as *Persicaria chinensis)*, was the first investigated heterostylous species [24].

Heterostyly is a floral polymorphism that is defined as a reciprocal placement of anthers ant stigmas in two or three floral morphs (distyly or tristyly respectively) of a species [25, 26, 27]. The long styled morph have stigma(s) positioned above the anthers, small pollen grains and high stigmatic papillae, whereas, the short-styled morph showing an anthers placed above the stigmatic surface, larger pollen grains, and short stigmatic papillae [25].

A growing body of literature has examined the distyly which has been proved in several *Polygonum* species such as *P*. *jucundum* Meisn [24]. The value of anther, ovary, seed, and achene anatomy in the *Polygonaceae* has been realized by several workers who have used it in their taxonomic treatment [28]. In addition, same studies mostly focusing on Chinese species discuss the heterostylous polymorphism in *Polygonum jucundum* Meisn. [29, 30, 31]. According to the data available in the literature, no information is currently available on the floral development of the *Polygonum* species in North Africa. In addition, there is a lack of knowledge of the floral vascularization and the organogenesis in this genus. In this work, we analyzed the flower, seed and fruit development of *Polygonum equisetiforme* Sm., *P*. *maritimum* L. and *P*. *aviculare* L. from Tunisia. The floral vascularisation characteristics were examined in *P*. *equisetiforme* flowers. The objectives of the present study were to reveal a comparative morpho-anatomical floral analysis of the distylous dimorphism, to anatomically characterize the tepal, anther, ovary, seed, and fruit development of these species and to develop new knowledge on the floral vascularization of *Polygonum* sect. *Polygonum*.

## Materials and methods

### Collected material

Flower buds, mature flowers, and fruits of *P*. *equisetiforme* var. *graecum* Meisn., *P*. *equisetiforme* var. *peyerinhoffi* Batt. & Maire and *P*. *maritimum* were collected from natural habitats of Djerba (Tunisia) in three sites (33°47’49”N, 11°02’51”E), (33°48’41”N, 11°02’38”E) and (33°43’14”N, 10°59’02”E), respectively. *P*. *equisetiforme* var. *peyerinhoffi* Batt. & Maire is characterized by a very numerous upright stems leafy at the base only, forming dense bushes. However, *P*. *equisetiforme* var. *graecum* Meisn. has hard steep stems more or less spreading or decumbent [8]. The plant material of *P*. *aviculare* was harvested from natural habitats of Nabeul Province, Tunisia (36°30’64”N, 10°39’14”E). All collections were carried out during May to July 2017 and 2018. All the plant material was provided by the “Laboratoire des écosystèmes pastoraux et valorisation des plantes spontanées et des micro-organismes associés, Institut des Régions Arides (IRA) Médenine, Tunisia”. Since *P*. *equisetiforme*, *P*. *aviculare* and *P*. *maritimum* are not a protected species and as the plants collecting focused on public rights of way, no a collecting permit required, nor was a specific permission needed. Vouchers of examined *Polygonum* species are deposited in the seed bank of the Laboratoire d'Ecologie Pastorale at the Institut des Régions Arides, Médenine, Tunisia (IRA).

### Floral and seed anatomy

Collected materials were fixed in freshly prepared FAA (formaldehyde: glacial acetic acid: 70% ethanol 5:5:90 by volume) overnight at room temperature and preserved in 70% ethanol. After washing with 0.1 M phosphate buffer (pH 7.4), they were dehydrated by passage through a tertiary butyl alcohol series (15–100%) and embedded with warm (56–58°C) paraffin. Histological blocks were prepared from each embedded material and then cut in 10–15 μm sections with a Sakura SRM200 rotary microtome (Sakura Accu-Cut SRM, Japon) with disposable blades, then stuck onto histological slides and dried using an electric slide warmer for 12 h. Dried slides were stained with 0.1% Toluidine blue O for 60–90 s, rinsed with running water, and again dried with an electric slide warmer for more than 6 h to remove water. The stained slides were then mounted with synthetic Canada balsam (Biopur) and the observations are performed under a light microscope (Leitz, Germany), and photographed with an attached camera system (Leica, Japon).

### Floral measurements

For observations and measurements on floral organs buds at similar stages of development, a hundred buds from each morph were sampled from 20 plants per style morph [30]. The tepals, stamens, and ovary were carefully removed from each bud, observed using a Leica MS5 stereomicroscope and the images of floral organs were captured using a Leica digital camera. The filament length, anther length, anther height, pollen size, ovary length, style length, and stigma height were measured from these images by using the metrical software Image J [32]. Details of pollen morphology were based on the measurements of 20 grains. The equatorial diameter (E) and polar axis (P) were determined from the images taken with the Leitz microscope equipped with a camera system. We used one-way analysis of variance ANOVA to analyze flower traits individually, with correction by sequential Bonferroni.

## Results

### Flower structure and development

**Fig 1:**
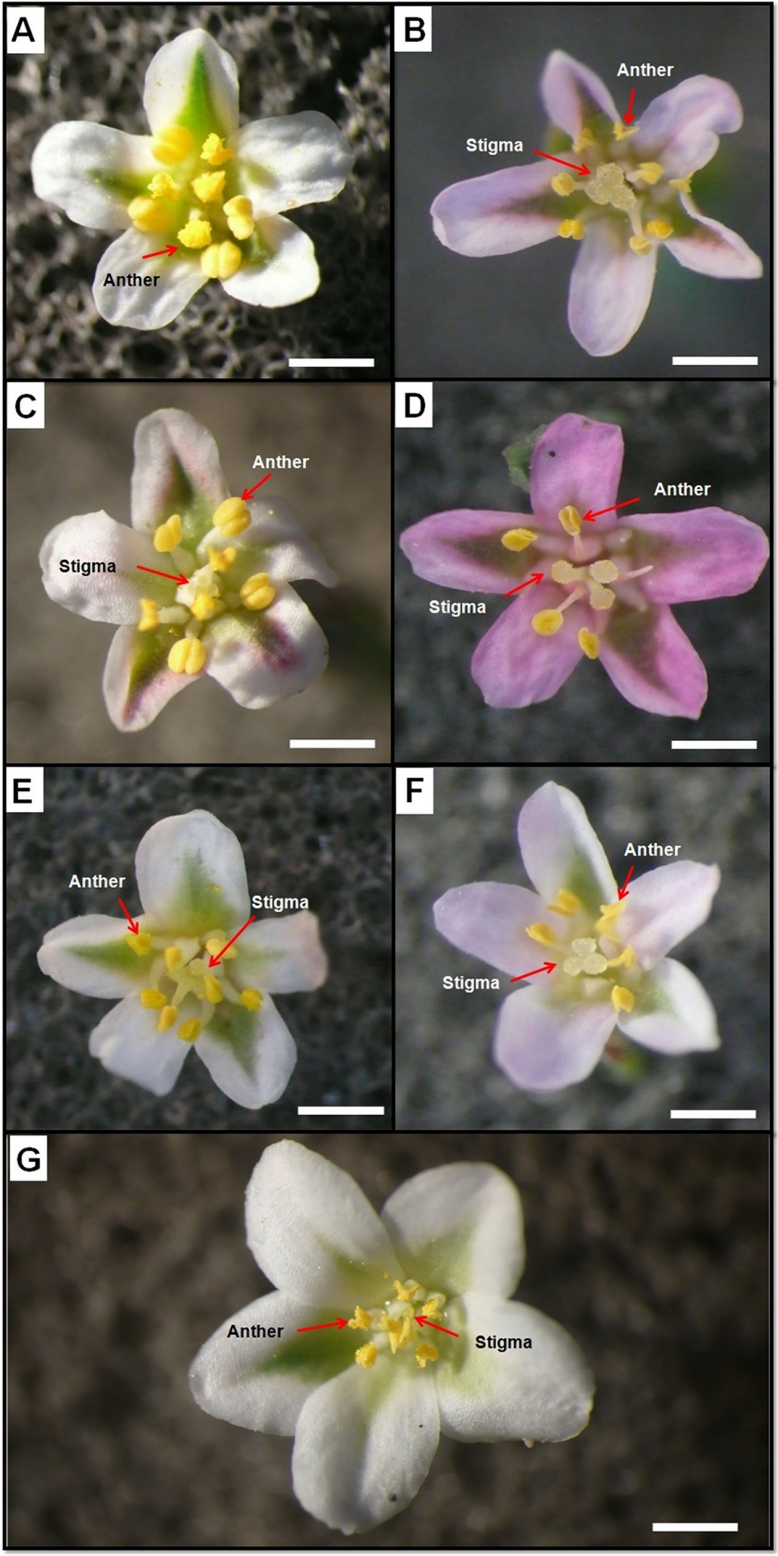
Flower morphology. Flowers of the long (LS) and short (SS) style morphs of *P*. *equisetiforme* var. *graecum* (a: SS, b: LS), *P*. *equisetiforme* var. *peyerinhoffi* (c: SS, d: LS), *P*. *aviculare* (e: SS, f: LS), and *P*. *maritimum*, (g). Scale bars = 1 mm.

*Polygonum maritimum* presented leafy inflorescences, flowers are in small axillary clusters, and leaves are coriaceous. *Polygonum aviculare* showed leafy inflorescences, solitary flowers, axillary, or in small axillary clusters with 3–5 flowers; bracts leaf-like, longer than the flowers. However, *Polygonum equisetiforme* presented spiciform inflorescences, terminal, slender, not leafy, and leaves are not coriaceous. The flowers have five oblong tepals, two outers, one intermediate, and two inner tepals. The outer and intermediate ones are oblong-elliptic while, the inner is smaller and slightly angular around the main vein. Results showed that the outer and inner tepals are significantly longer and wider in *P*. *maritimum* while they have similar dimensions in *P*. *aviculare*, *P*. *equisetiforme* var. *graecum* and var. *Peyerinhoffi* (Fig 1, Table 1 and Table 2).

**Table 1:** Floral dimensions (mean ± standard deviation) of the long styled (LS) and short styled (SS) flowers of *Polygonom* L. species. a: *P*. *equisetiforme* var. *graecum*, b: *P*. *equisetiforme* var. *peyerinhoffi*, c: *P*. *aviculare*, d: *P*. *maritimum*.

Floral traits	a		b		c		d
	LS-morph	SS-morph	LS-morph	SS-morph	LS-morph	SS-morph	
Inner tepal length (mm)	1.215±0.081	1.209±0.094	1.212±0.058	1.220±0.049	1.251±0.102	1.242±0.080	2.233±0.124
Outer tepal length (mm)	0.601±0.034	0.596±0.041	0.613±0.052	0.603±0.039	0.620±0.057	0.646±0.071	1.502±0.105
Style length (mm)	2.791±0.114	1.193±0.097	2.783±0.125	1.206±0.099	2.604±0.131	1.153±0.096	0.963±0.051
Stigma height (mm)	0.326±0.032	0.260±0.029	0.336±0.041	0.251±0.017	0.330±0.027	0.283±0.035	0.262±0.022
Stigma widh (mm)	0.473±0.037	0.361±0.042	0.486±0.058	0.346±0.028	0.520±0.071	0.393±0.042	0.341±0.034
Ovary height (mm)	1.503±0.120	1.508±0.134	1.530±0.098	1.504±0.085	1.396±0.108	1.403±0.113	1.510±0.125
Ovary widh (mm)	0.903±0.058	0.891±0.064	0.883±0.049	0.902±0.067	0.786±0.045	0.776±0.051	1.143±0.082
Stamen height (mm)	3.093±0.218	3.876±0.328	3.114±0.302	3.895±0.297	2.876±0.328	3.677±0.406	3.139±0.307
Filament length (mm)	2.584±0.324	3.384±0.491	2.581±0.375	3.396±0.534	2.281±0.281	3.275±0.359	2.726±0.289
Anther height (mm)	1.180±0.158	0.803±0.107	1.145±0.098	0.895±0.124	0.757±0.076	0.791±0.068	0.607±0.054
Anter widh (mm)	0.924±0.051	0.599±0.038	0.844±0.075	0.653±0.057	0.569±0.062	0.580±0.045	0.377±0.042
Polen diameter (μm)	31.50±1.240	34.19±1.420	30.83±1.380	32.54±1.480	28.36±1.370	33.51±1.590	28.15±1.400

**Table 2:** P-values of the statistical analyses (ANOVA) related to floral morphology of *P*. *equisetiforme* var. *graecum*, *P*. *equisetiforme* var. *peyerinhoffi*, *P*. *aviculare* and *P*. *maritimum*.

Floral traits	Test between subjects effects			
	n	Morphs	Species	Morphs × Species
Inner tepal length (mm)	20	0.010	0.915	0.954
Outer tepal length (mm)	20	0.036	0.661	0.307
Style length (mm)	20	< 0.0001	< 0.0001	0.008
Stigma height (mm)	20	0.311	< 0.0001	0.154
Stigma widh (mm)	20	0.019	< 0.0001	0.688
Ovary height (mm)	20	< 0.0001	0.487	0.245
Ovary widh (mm)	20	< 0.0001	0.935	0.546
Stamen height (mm)	20	< 0.0001	< 0.0001	0.888
Filament length (mm)	20	< 0.0001	< 0.0001	< 0.0001
Anther height (mm)	20	< 0.0001	< 0.0001	< 0.0001
Anter widh (mm)	20	< 0.0001	< 0.0001	< 0.0001
Polen diameter (μm)	20	0.007	< 0.0001	0.011

The pattern of floral variation demonstrates that two out of the three studied species are distylous characterized by the reciprocal placement of stigmas and anthers in two floral morphs. The flowers possess eight stamens situated at the base of the tepals and arranged in one outer whorl composed of five stamens and inner ones of three stamens (Fig 1). The stamens of both whorls are free. Observations of *P*. *equisetiforme* (var. *graecum* and *peyerinhoffi*), and *P*. *aviculare* revealed the occurrence of two morphs as distylous species: short style morph (SS) and long style morph (LS) known as thrum type and pin-type respectively. In contrast, *P*. *maritimum* have homostylous flowers (Fig 1). In *P*. *equisetiforme* (var. *graecum* and *peyerinhoffi*), and *P*. *aviculare*, the color of the flowers varied from whitish to pink. In detail, the pin flower of *P*. *equisetiforme* var. *graecum* and *P*. *aviculare* have a whitish color (1A and 1E) and the thrum ones are light pink (Fig 1B and 1F). In *P*. *equisetiforme* var. p*eyerinhoffi*, the LS flower is of smooth to light pink color while the SS one is dark pink whatever the flower age (Fig 1C and 1D). For *P*. *maritimum* we always found white flowers (Fig 1G). The style length of LS morph was significantly (P <0.0001) longer than that of the SS morph. The long style length is higher in *P*. *equisetiforme* var. *graecum* and var. *peyerinhoffi* compared with *P*. *aviculare*. However, the lowest value of style length was measured in *P*. *maritimum* (0.96 ± 0.02 mm). The difference in stigma height and width between LS and SS flowers was very significant (Fig 1). Thus, the stigma width of LS morph stands approximately 1.25, 1.3, and 1.4 fold greater than that of the SS morph in *P*. *equisetiforme* var. graecum, *P*. *equisetiforme* var. *peyerinhoffi*, and *P*. *aviculare*, respectively. Moreover, the stigma height of LS morph is unchanged in the studied species, whereas the SS morph of *P*. *aviculare* indicated the highest value. Our results showed that the ovary height and width was unchanged by the style morphs. Among the species, *P*. *aviculare* has the lower size in both morphs while the higher one was observed in *P*. *maritimum*. The filament length and the stamen height were significantly greater in the SS morph compared to LS morph in *P*. *aviculare*, in *P*. *equisetiforme* var. *graecum* and var. *peyerinhoffi*. For these floral parameters, the lowest values were found in *P*. *aviculare* and in *P*. *maritimum*. For *P*. *equisetiforme* there is no significant difference between the varieties. The anther length and width of LS morphs in both varieties of *P*. *equisetiforme* was significantly (P <0.0001) longer than that of the SS morphs while they were unchanged in the flowers morphs in *P*. *aviculare*. The results indicated no significant difference for these parameters between var. *graecum*, and var. *peyerinhoffi* of *P*. *equisetiforme*. The higher anther length and width measured in *P*. *equisetiforme* var. *graecum* flowers were closer to 1.18 and 0.92 mm respectively, whereas the lowest values occurred in *P*. *maritimum*. Pollen sizes of the two morphs are different (P <0.0001). The long-styled flowers produce significantly smaller pollen than the short-styled ones. However, the pollen diameter of *P*. *maritimum* was the lowest while it was higher in the SS morph of *P*. *equisetiforme* with not significant difference between the varieties.

**Fig 2:**
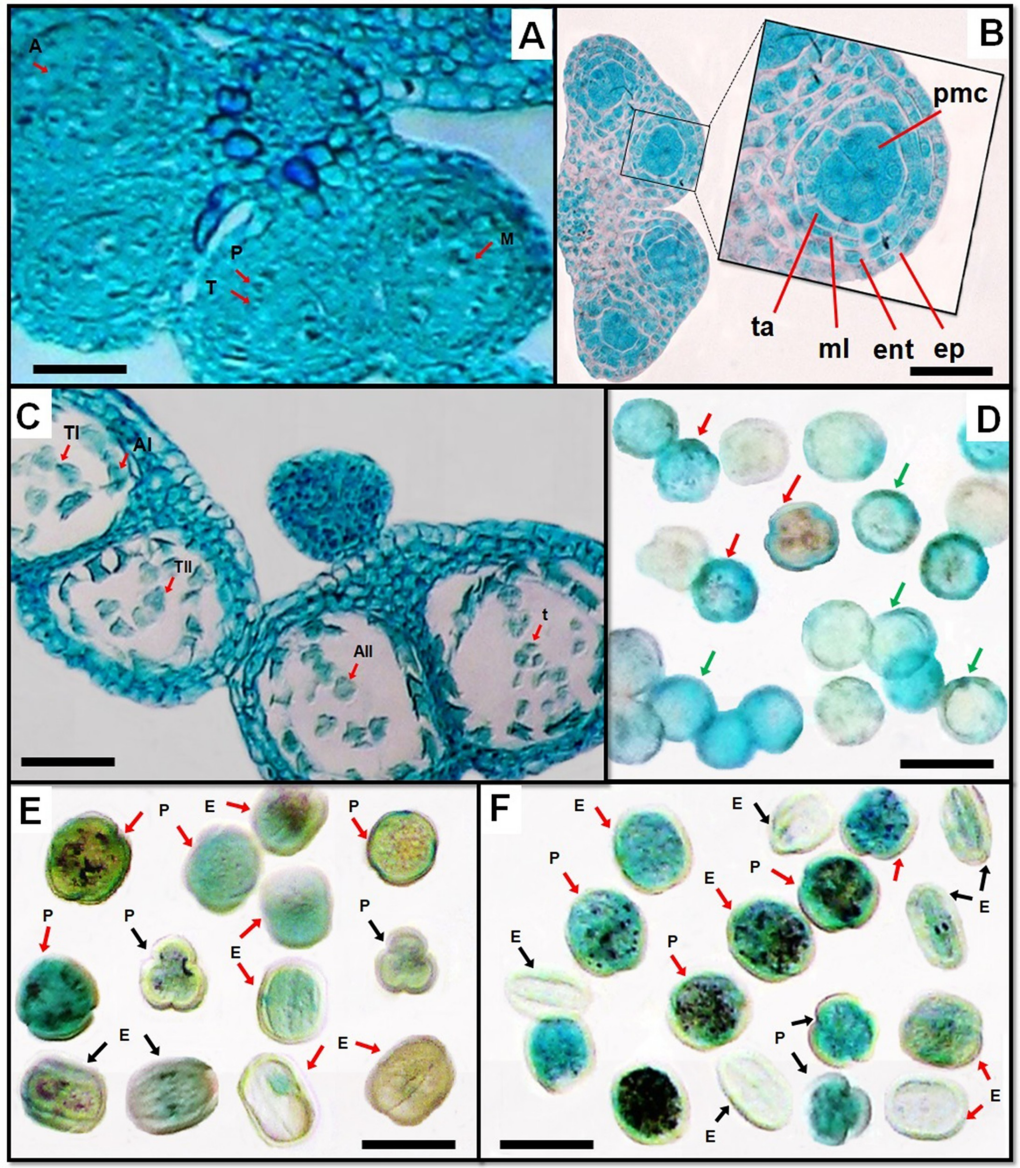
Anther wall formation, microsporogenesis, microgametogenesis and morphology of pollen grains of *Polygonum*. A, Cross-sectional view of anther, showing a row of secondary sporogenouscells gave rise to a mass of microspore mother cells by several mitotic divisions (arrow). B, Anther wall formation. C, Microsporocytes cells at anaphase I, telophase I, anaphase II and telophase II (arrow). Pollen morphology of *P*. *maritimum* (D), *P*. *aviculare* (E) and *P*. *equisetiforme* (F) under light microscope. Pollen grains are rounded-trilobed with deep long colpi in polar view and prolate in equatorial view (black arrow). Pollen grains of circular shape with shallow long colpi in polar view (red arrow). Pollen grains of circular polar outline and spheroidal to prolate-spheroidal equatorial view (green arrow). Abbreviations: A, anaphase; T, telophase, P, prophase; M, metaphase; AI, anaphase I; AII, anaphase II, TI, telophase I; TII, telophase II; t, tetrads; ent, endothecium; ml, middle layers; ta, tapetum; pmc, pollen mother cells; E, equatorial view; P, polar view. Scale bars: A, B and C = 400 μm; D, E and F = 35 μm.

Microscopic observation showed that the young anthers are tetrasporangiate connected to the interface tissue (Fig 2). At the beginning of the development of the anther, the pollen sac comprises a group of archesporial cells whose division and differentiation generates wall layers. Before maturation, the anther wall is formed by four cell layers, viz. epidermis, endothecium, middle layers and tapetum (Fig 2B). Towards the inside in the middle part of the pollen sac, we find a large group of cells with dense cytoplasm and visible nuclei which are the pollen mother cells (PMC) who are surrounded by the tapetum. The middle layer and tapetum degenerated during meiosis while the epidermis and endothecium layers were observed throughout anther development. Indeed, the walls of the mature anther consist of thick fibrous endothecium cells and the papillate epidermis (Fig 2C).

In the stamen primordia, the archesporial cells differentiated and divided periclinally to form outer primary parietal cells and inner primary sporogenous cells. The primary parietal cells divided repeatedly to form a subepidermal endothecium, whereas inner cells produce the middle layer cells and the tapetum. In addition, inner primary sporogenous cells divided again periclinally to form secondary sporogenous cells, which gave rise to a mass of microspore mother cells by several mitotic divisions (Fig 2A). Later, the microsporocytes progressed to meiosis I (prophase I, metaphase I, anaphase I and telophase I) and II (prophase II, metaphase II, anaphase II and telophase II) and the four cell walls of the anther were formed (Fig 2B). Also, a microspore tetrad with mostly tetrahedral shape was produced.

### Flower anatomy and vasculature

**Fig 3:**
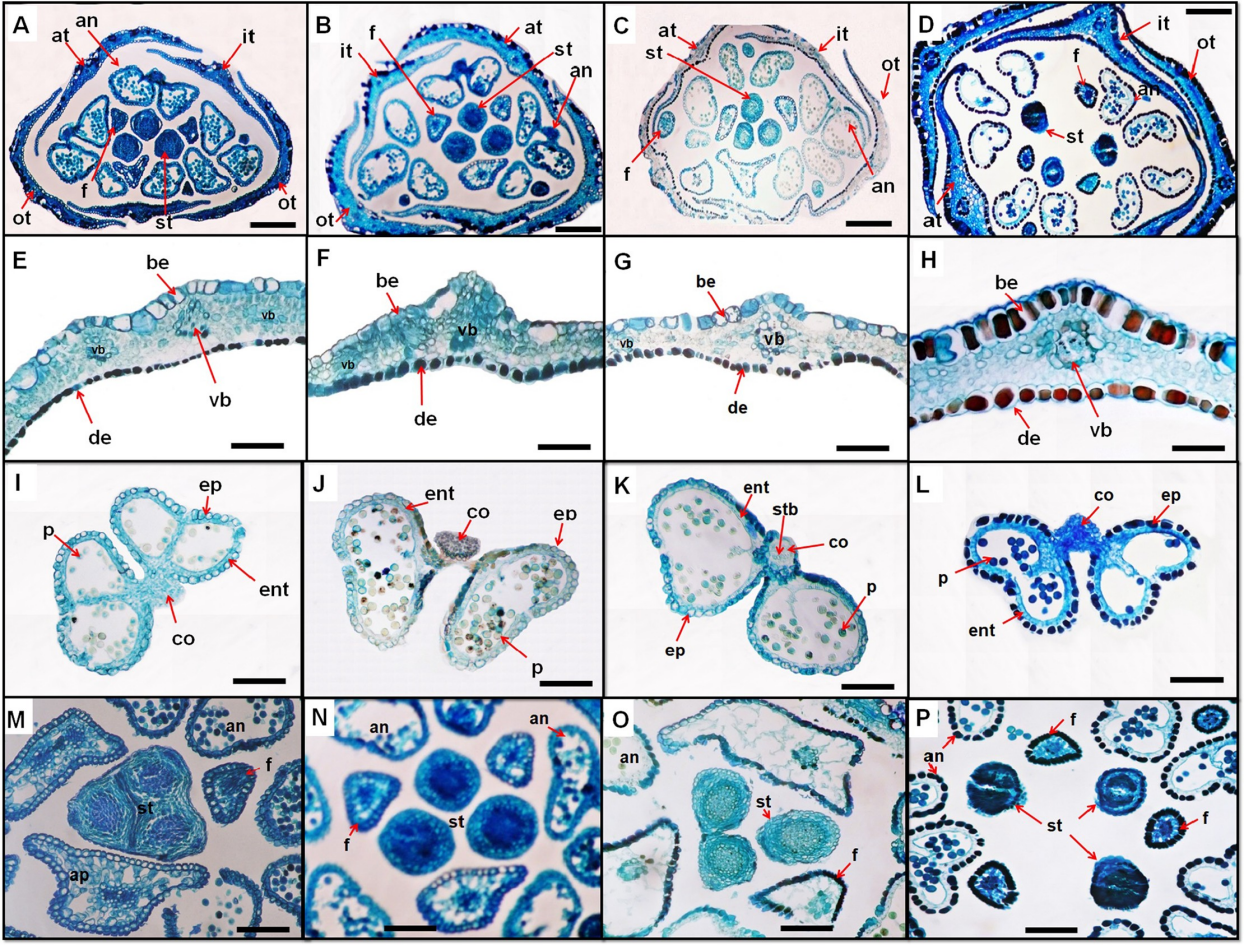
Anatomical aspects of the flowers. *P*. *equisetiforme* var. *graecum* (A, E, I and M), *P*. *equisetiforme* var. *peyerinhoffi* (B, F, J and N), *P*. *aviculare* (C, G, K and O), and *P*. *maritimum* (D, H, L and P) under light microscope. Flower buds cross section (A, B, C and D). Tepal detail (E, F, G and H). Detail of mature anther (I, J, K and L). Detail of the free portion of the filament and style-stigma (M, N, O and P). Abbrevations: an, anther; be, abaxial epidermis; co, connective; de, adaxial epidermis; ep, epidermis; ent, endothecium; f, filament; it, inner tepal; ot, outer tepal; at, alternate tepal; st, stigma; stb, stamen vascular bundle; p, pollen; vb, vascular bundle. Scale bars: A, B, C and D = 150 μm; E, F, G, H, I, J, K, L, M, N, O and P = 500 μm.

The tepal epidermis is similar in the three species investigated, consisting of irregular, elongated or rectangular cells with sinuate outline. The cuticle is often deeply and irregularly ridged. In cross-section, the abaxial epidermis has larger cells, much wider lumen and thin walls. The adaxial epidermis has much smaller, thin-walled cells. One main vein is present in each tepal. The secondary veins are frequent especially in *P*. *equisetiforme* and *P*. *aviculare* with smaller sizes especially in the last species (Fig 3E–3H). In *P*. *maritimum* secondary veins are much less common. Stomata are usually present in the abaxial surface in *P*. *equisetiforme* and *P*. *maritimum* whereas it was observed on both the abaxial and adaxial surface of the tepal in *P*. *aviculare*.

The flowers possessing eight stamens situated at the base of the tepals and arranged in one outer whorl composed of five stamens and an inner one of three stamens (Fig 1). The stamens of both whorls are free. All filaments are flattened with thickened base in the inner stamens. Anatomically, the filaments are formed by one layered epidermis with isodiametric cells and a parenchymatous mesophyll composed of rounded cells. The vasculature consists of one central vascular bundle (Fig 3M–3P). The anthers in the three studied species are dithecal, tetrasporangiate, basifixed, with longitudinal dehiscence. The mature anther wall consists of an epidermis and endothecium (Fig 3I–3L). The epidermis has thickened rounded cells that are larger in the stomium region. The connective is formed by an epidermis with much smaller cells, parenchyma, and the one staminal bundle. The endothecium is located around each pollen sac, and its cells are thin-walled elongated quadrangulate in cross-section. The epidermis and endothecium cells width were higher in *P*. *maritimum* and the lowest endothecium width was observed in *P*. *aviculare*.

The mature pollen grains observed in the *Polygonum* L. *section Polygonum* studied species are tricolporate showing variation in pollen shape and size. The shape of pollen in *P*. *aviculare* and *P*. *equisetiforme* are the most similar. Indeed, in polar view, the majority of pollen are of circular shape with shallow colpi but some others are of circular-trilobate shape with deep colpi. In equatorial view, pollen grains are predominantly prolate. In contrast, in *P*. *maritimum*, the pollen grains are of circular shape rarely with shallow colpi in polar view and spheroidal to prolate-spheroidal in equatorial view (Fig 2D and 2F). The average diameter of pollen grainswas in the range of 28.2–34.5 / 24.6–27.5 μm. *P*. *maritimum* (27.9/24.6 μm) appeared to be the smallest in pollen diameter while *P*. *equisetiforme* (34.5/25.1 μm) was the largest (Table 3). The ratio P/E (polar axis/equatorial diameter) varied from 1.13–1.37, minimum in *P*. *maritimum* and maximum in *P*. *equisetiforme*. An exine was clearly visible in all species, quite thick in *P*. *maritimum* (3 μm) followed by *P*. *aviculare* (2.6 μm) while relatively thin exine was recorded in *P*. *equisetiforme* (1.9 μm). In contrast, the exine pattern under light microscope is invisible.

**Table 3:** Morpho-anatomical characteristics of the flowers of *Polygonom* L. species. a: *P*. *equisetiforme* var. *graecum*, b: *P*. *equisetiforme* var. *peyerinhoffi*, c: *P*. *aviculare*, d: *P*. *maritimum*.–absence, + presence.

Floral traits	a	b	c	d
*Tepals*				
Five tepals	+	+	+	+
Tepal color varies from whitish to light pink	+	-	+	-
Tepal color varies from light to dark pink	-	+	-	-
White tepals	-	-	-	+
Epidermis cells are elongated or rectangular cells with sinuate outline	+	+	+	+
Stomata on abaxial and adaxial surface	-	-	+	-
Stomata on the abaxial surface	+	+	-	+
Tepal vascularisation with one main vein	-	-	-	+
Tepal vascularisation with one main vein and secondary vein	+	+	+	-
*Androecium*				
Eight free stamens arranged in one outer whorl with five stamens and inner one of three stamens	+	+	+	+
Filaments with parenchymatous mesophyll	+	+	+	+
Anthers bithecal and tetrasporangiate	+	+	+	+
The mature anther wall consists of epidermis and endothecium	+	+	+	+
Thicker epidermis and endothecium	+	+	-	+
Thinner endothecium	-	-	+	-
Smaller size with P/E (27.9/24.6 μm)	-	-	-	+
Larger size with P/E (34.5/25.1 μm)	+	+	-	-
Larger size with P/E (33.0/27.5 μm)	-	-	+	-
Thicker exine (3 μm)	-	-	-	+
Thicker exine (2.6 μm)	-	-	+	-
Relatively thinner exine (1.9 μm)	+	+	-	-
Higher P/E ratio (1.37)	+	+	-	-
Intermediate P/E ratio (1.2)	-	-	+	-
Lower P/E ratio (1.13)	-	-	-	+
Pollen grains tricolporate	+	+	+	+
Pollen grains are rounded-trilobed with deep long colpi in polar view and prolate in equatorial view	+	+	+	-
Pollen grains of circular shape with shallow long colpi in polar view and also prolate in equatorial view	+	+	+	-
Pollen grains are of circular polar outline and spheroidal to prolate-spheroidal equatorial view	-	-	-	+
*Gynoecium*				
Three free styles connate at base with three capitate stigmas	+	+	+	+
Distylous	+	+	+	-
Monostylous	-	-	-	+
Ovary unilocular	+	+	+	+
Ovary monovulate	+	+	+	+
Ovule anatropous	+	+	+	+
Basal placentation	+	+	+	-
Apical placentation	-	-	-	+
*Seed coat*				
Thicker exotesta with larger thick-walled cells	-	-	-	+
Exotesta with less large rectangular cells	+	+	+	-
Mesotesta with smaller thin-walled cells	+	+	+	+
Endotesta with elongated thin-walled cells	+	+	+	-
Endotesta with rounded thin-walled cells	-	-	-	+
Tegmen with two-layer radially elongated cells	+	+	+	+
*Fruit*				
Fruit pericarp with exocarp, mesocarp, and endocarp	+	+	+	-
Thicker exocarp with undulated anticlinal walls and a broad lumen with dendritic branching towards the periphery	-	-	+	-
Exocarp with less undulating anticlinal walls and narrow branching lumen	+	+	-	-
Exocarp with straight anticlinal walls and broad lumen	-	-	-	+
Mesocarp with 2−3 layers of flattened cells	+	+	+	+
Fruit are trigonous with ovate-lanceolate shape	+	+	+	+
Fruit is dark brown with three subequal concave sides	+	+	-	-
Fruit is black with highly concave faces	-	-	+	-
Fruit is black with slightly concave faces	-	-	-	+
Acene surface is shiny and smooth	-	-	-	+
Achene surface is striate-tubercled	+	+	+	-
Smaller size with L**×**W (1.9 x 1.5 mm)	-	-	+	-
Moderate size with L**×**W (2.85 x 1.46 mm)	+	+	-	-
Larger size with L**×**W (4.05 x 2.98 mm)	-	-	-	+

**Fig 4:**
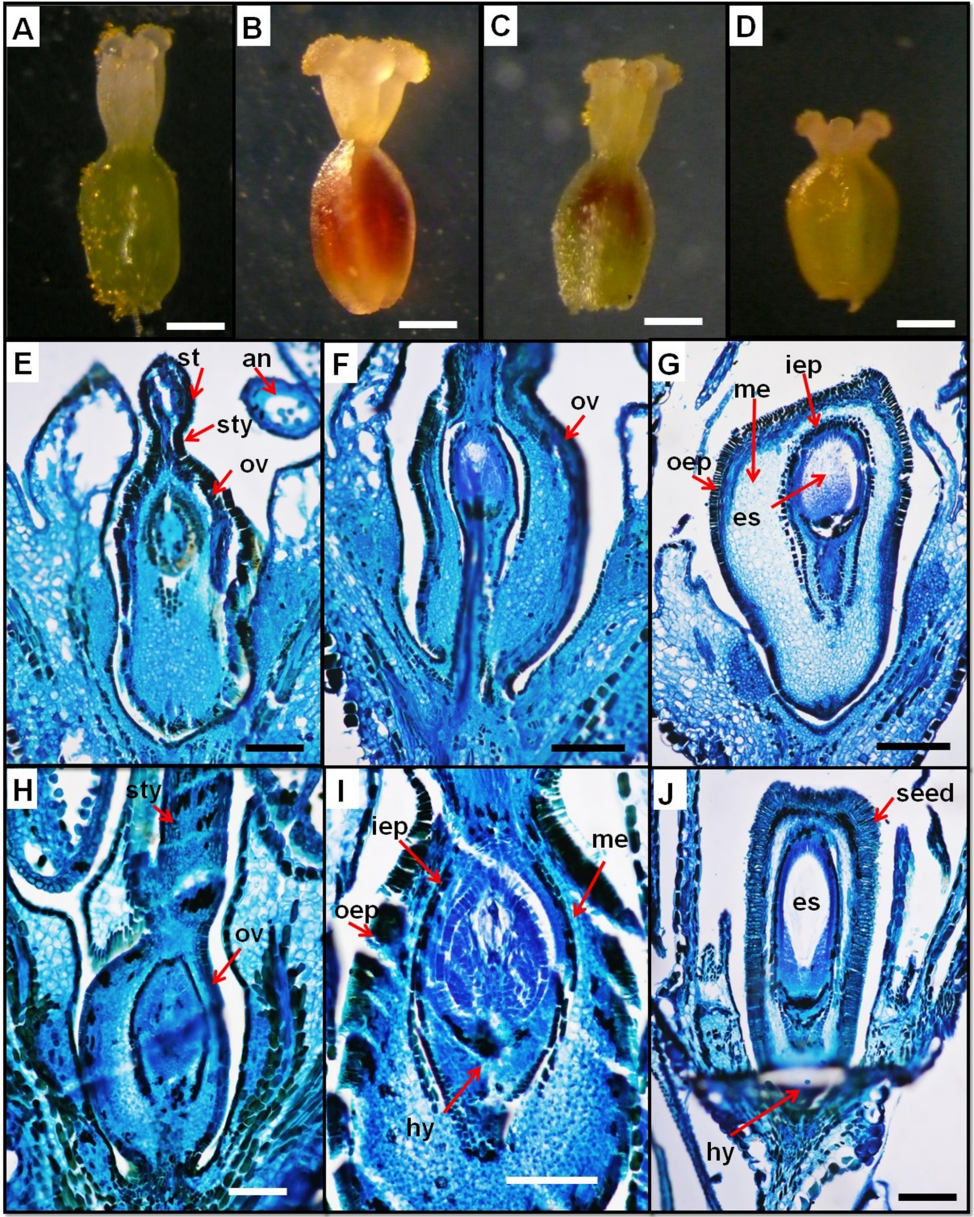
Anatomical aspects of the ovary. *P*. *equisetiforme* var. *graecum* (A), *P*. *equisetiforme* var. *peyerinhoffi* (B), *P*. *aviculare* (C), and *P*. *maritimum* (D) under stereomicroscope. Longitudinal section of the flower and young fruit in *P*. *maritimum* (E, F and G) and *P*. *equisetiforme* (H, I and J). Abbreviations: hy, hypostase; iep, inner epidermis; oep, outer epidermis; me, mesophyll, ov, ovary; sty, style; st, stigma; es, embryo sac. Scale bars: A, B, C and D = 800 μm; E, F, G, H, and J = 400 μm; I = 300 μm.

The gynoecium is differentiated into stigma, style, and ovary (Figs 3 and 4). The ovary is uniovulate and trigonous, with an ovate-circular shape. The ovule is anatropous with basal placentation in *P*. *equisetiforme* and *P*. *aviculare* and apical placentation in *P*. *maritimum* (Fig 4). In the three studied species, the ovary is prolonged by 3 moderately short styles, anatomically formed by an epidermis composed of oblong-elliptic cells covered by a thin cuticle. Towards the inside, the parenchyma is made up of rounded cells in cross-section. The central zone of the style is composed of smaller thick walled parenchymatous cells forming a compact mass of transmission tissue (Fig 4E, 4F and 4J). This tissue is strongly stained, indicating its secretory activity. The vasculature is ensured by collateral vascular bundles crossing the style and branching at the base of the stigmatic lobes. These styles are basally fused with three capitate stigmas.

**Fig 5:**
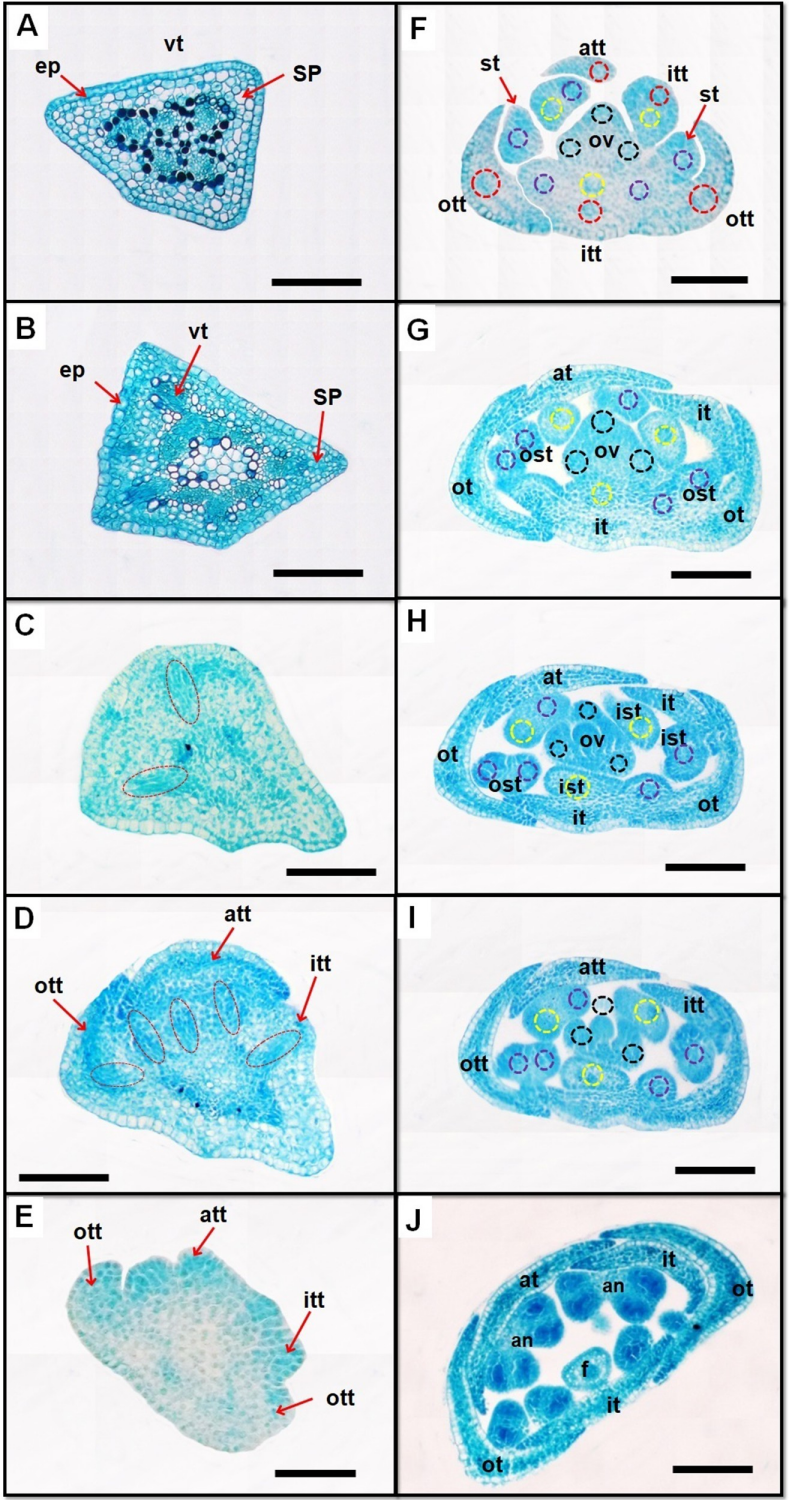
Floral vasculature. *P*. *equisetiforme* in transverse sections of developing flowers (A, B, C, D, E, F, G, H, I and J). Abbreviations: ep, epidermis; vt, vascular trace; SP, spangy parenchyma; att, alternate tepal trace; ott, outer tepal trace; itt, inner tepal trace; st, stamen trace; ost, outer stamen trace; ist, inner stamen trace; yellow circle, inner stamen trace; purple circle, outer stamen trace; red circle, tepal trace; black circle, ovary trace. Scale bars = 50 μm.

Cross sections show that the floral pedicel of *Polygonum* studied species is sub-trapezoidal, formed by epidermis, cortex and five collateral bundles arranged in a ring (Fig 5A and 5B). This central stele will diverge to extend in many vascular bundles and give rise to the vasculature of all flower parts (tepals, stamens and the ovary). At first two traces depart from the main stele simultaneously diverging into two corners (Fig 5C), followed by three more traces (Fig 5D).

### Seed and fruit development

**Fig 6:**
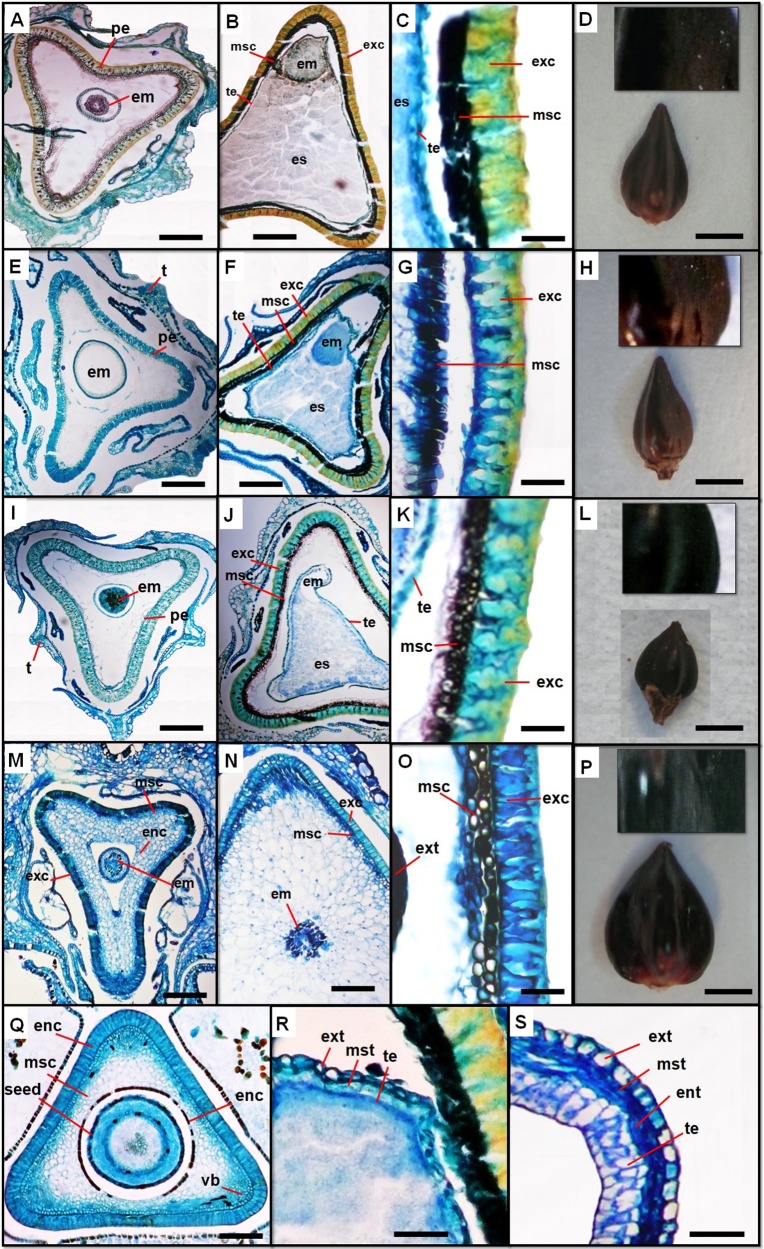
Morphological and anatomical aspects of fruit. *P*. *equisetiforme* var. *graecum* (A, B, C and D), *P*. *equisetiforme* var. *peyerinhoffi* (E, F, G and H), *P*. *aviculare* (I, J, K and L), and *P*. *maritimum* (M, N, O and P) under light microscope. Transversal section of premature fruit (A, E, I, M and Q). Transversal section of mature fruit (B, F, J, N and R). Transversal section of mature fruit wall (C, G, K, O and S). Mature fruits morphology under light microscope (D, H, L, and P). Mature seed coat (R). The young seed coat (S). Abbreviations: an, anther; em, embryo; enc, endocarp; es, embryo sac; exc, exocarp; msc, mesocarp; oep, outer epidermis; oi, outer integument; pe, perisperm; sc, seed coat; vb, vascular bundle; enc, endocarp; ent, endotesta; ext, exotesta; mst, mesotesta; te, tegmen. t, tepal. Scale bars: A, B, E, F, I, J, M and N = 400 μm; C, G, K and O = 80 μm; D, H, I and P = 1000 μm; Q = 250 μm; R and R = 100 μm.

Normally, the outer and inner epidermis and mesophyll of the carpel will differentiate into three components of the pericarp which are: exocarp, mesocarp, and endocarp. In young fruit of the studied species, the exocarp is composed of a single layer of rectangular cells, with nearly straight thick anticlinal and periclinal walls and vast cavities. The structure of the exocarp changes during maturation, with highly lignified narrow cells and irregularly undulate anticlinal walls. In *P*. *aviculare*, the exocarp is thicker compared to the two other species, the anticlinal walls are very twisted and the cells cavities are almost rectangular with several ramifications on the sides. The periclinal wall contains some projections (verrucae) on the surface (Fig 6I–6K). For *P*. *equisetiforme*, the mature exocarp consists of smaller rectangular cells with narrow cavities (Fig 6A–6G). The anticlinal wall is less undulating with several verrucae. A thinner exocarp is observed in the mature fruit of *P*. *maritimum* characterized by the absence of verrucae on the surface, with narrow rectangular cells, straight anticlinal walls and vast cavities (Fig 6M–6O). During fruit development, the mesocarp cells become irregularly shaped by losing the protoplasts, increasing the intercellular space. At the external mesocarp level, the cross sections showed the presence of vascular bundles, attached with the internal exocarp (Fig 6Q). In mature fruit, the mesocarp is characterized by 2−3 layers of compressed flattened cells (Fig 6C, 6G, 6K and 6O). In the young fruit, the endocarp is formed by uniseriate cells; likewise, during maturity, these cells lose protoplasts and become very elongated. In the studied species, the endocarp has completely collapsed in mature fruit (Fig 6). The young seed coat is composed of the following compartments: the external one, the exotesta, consists of uniseriate large thick-walled cells (Fig 5S). The mesotesta is formed by 2 layers of smaller thin-walled cells, and an endotesta is formed by a single layer of elongated thin-walled cells. The tegmen is composed of a single or two layers of radially elongated cells that are rich in protoplasts. In the mature seed coat, a single layer of rectangular cells elongated tangentially forming the exotesta. The mesotesta is composed by one or two layers of much-flattened cells also, the endotesta is reduced to a single layer of elongated and small width cells (Fig 6R).

The fruits of the studied *Polygonum* species are trigonous with ovate-lanceolate shape, often unequally triangular with one side broader than the other two, with a short stipe and long beak (Fig 6 and Table 3). The mature fruit is dark brown with three subequal concave sides in *P*. *equisetiforme* (Fig 6D and 6H), black with highly concave faces in *P*. *aviculare* (Fig 6L) and black with slightly concave faces in *P*. *maritimum* (Fig 6P). The external achene surface is shiny and smooth in *P*. *maritimum*. On the contrary, it is striate-tuberculate in the other studied species. The undulating anticlinal wall of the exocarp cells give rise to the observed tubercles. While there are few equidistant tubercles covering the achene of *P*. *equisetiforme*, they are arranged in longitudinal rows in *P*. *aviculare*. The achene sizes (length x width) was larger in *P*. *maritimum* (4.05 x 2.9 mm) followed by *P*. *equisetiforme* (2.85 x 1.46 mm) while smaller achenes (1.9 x 1.5 mm) were found in *P*. *aviculare* (Table 3).

## Discussion

The present study allowed us to provide essential information on the morpho-anatomy and the flower vascularization in three species of the genus *Polygonum* sect. *Polygonum*. With these results, we contribute to the knowledge of these species. Our study revealed that *P*. *aviculare*, *P*. *equisetiforme* var. *graecum* and var. *peyerinhoffi* are distylous species with a polymorphism affecting the style and stamen height, tepal, pollen and seed size (Tables 1 and 2). However, we found only one morph having white styled flowers in *P*. *maritimum*. A change in the colour of the two flower morphs was observed in the two varieties of *P*. *equisetiforme* (Fig 1). There are some studies on the functions of dimorphism of the size of the tepal. Some researchers reported that short-style flowers had slightly larger tepals than long-style flowers [24, 27]. In Polygonaceae, the comparison of tepal size between morphs varies according to the studied taxa. We found no significant difference in tepal size between the different morphs of *P*. *equisetiforme* var. *graecum* and var. *peyerinhoffi* and *P*. *aviculare*, which was also observed in *P*. *hastato-sagittatum* Mak. [30], while a larger tepal length was observed in *P*. *jucundum* [24]. The two varieties of *P*. *equisetiforme* and *P*. *maritimum* are characterized by the presence of stomata on the abaxial surface. *P*. *aviculare* can be diagnosed by the occurrence of stomata on both the abaxial and adaxial epidermis of the tepals. The distribution of stomata on the tepals of all studied species may be explained by the effect the climatic and environmental factor in which these species occur, in fact, *P*. *aviculare* grow in superior semi-arid habitats, whereas *P*. *equisetiforme* and *P*. *maritimum* are found in dry habitats. As stomata promote water loss during gas exchange, plants with few stomata may be advantageous in drier environments.

In the studied *Polygonum* species, the stamens are free and arranged in outer and inner whorls, they have long flattened filaments and are independent of separate tepals. These observations are in agreement with the finding in *P*. *hastato-sagittatum* and *P*. *jucundum*, respectively [30]. In contrast, the filaments have little contribution to the anther height in other distylous species [33, 34]. The flower morphs of *P*. *equisetiforme* var. *graecum* and var. *peyerinhoffi* present a remarkable difference in the anther size with longer anthers in the SS flowers. This dimorphism was recorded in others distylous species such as *Psychotria nuda* (Cham. & Schltdl.) Wawra [35], and *Psychotria carthagenensis* Jacq. [36]. The anther showed one ephemeral middle layer, reported in many species such as *Chrysanthemum multicaule* Desf. [37], *Anchusa azurea* Mill., *Asperugo procumbens* L., *Cynoglossum glochidiarum* Wall., *C*. *lanceolatum* Forssk., *Lycopsis aroensis* L., *L*. *orientalis* L., *Myosotis sylvatica* Hoffm., *Rochefia stylaris* Boss., *Solenanrhus circinatus* Ledeb. [38] and *Swainsona formosa* (G. Don) Joy Thomps. [39]. The mature anther wall of *P*. *equisetiforme* and *P*. *maritimum* is characterized by well-developed endothecium also prevalent in many dicotyledonous species such as *Onobrychis schahuensis* Bornm [40], *Acca sellowiana* (O. Berg) Burret [41], and *Camellia japonica* L. [42]. On the contrary, recent studies have shown that the endothelium is not differentiated and or absent in the anther of *Camellia yunnanensis* var. *camellioides* (Hu) T.L.Ming [43] and of *Abelia tyaihyoni* Nakai [44].

Our study revealed that the three studied species of *Polygonum* have a similar morphology of pollen grains. They are all tricolporate showing variation only in shape and size. In agreement with previous findings in other *Polygonum* species showing that this genus has prolate to spheroidal pollen and the aperture is mostly tricolporate, rarely panto-hexacolporate with several types of exine ornamentation [45, 46]. The largest pollen size was observed in *P*. *equisetiforme*, while the smallest one in *P*. *maritimum*. In contrast, the latter showed the thicker exine while the lowest exine thickness was observed in the former. Previous papers recorded that the exine thickness varied from 1.00 μm (*P*. *sarobiense* Rech. f.) to 6.3 μm (*P*. *posumbu* Buch.-Ham. ex D.Don) [47, 48]. Besides pollen size, P/E value proved to be a useful character of systematic value. In the present study, the P/E ratio varied from 1.13 to 1.36 in agreement with pollen morphology of other *Polygonum* [48] and other Polygonaceae species such as *Fallopia convolvulus* (L.) Á. Löve and *F*. *dumetorum* (L.) Holub. [11].

The flowers of *Polygonum* are supplied by five fundamental vascular bundles. The tepal vasculature differs between the studied species, indeed, in *P*. *maritimum* the vasculature of each tepal is ensured by a single vascular bundle similarly to vasculature of sepal and petal of *Schefflera delavayi* (Franchet) Harmswhereas in *P*. *equisetiforme* and *P*. *aviculare* each tepal is supplied by one median vascular bundle and two lateral bundles as shown in *Schefflera heptaphylla* (L.) Frodin petal [49]. On the contrary, each stamen has a single vascular bundle as shown in *Buxus balearica* Lam. [50] and in 7 species of Asian *Schefflera* L. [49]. The observations of the floral vasculature in *P*. *equisetiforme* indicated that the stamen and tepal haven’t got a common primordial vasculature. A similar observation was made in *Eriogonum heracleoides* Torr., *E*. *umbellaius* Torr., *E*. *virgatum* Benth., and *Polygonum aviculare* L. [51], and in *Cadia purpurea* (G. Piccioli) Aiton [52]. In contrast, in *Pisum sativum* L. [53] and in nine species of *Cyclamen* [54] the stamens and petals are initiated in a common primordium. Simultaneously, the inner stamens are initiated followed by the inception of the gynoecium. This simultaneous emergence of the stamens is similar to those observed in *Plantago* L., *Aragoa* Kunth and *Heliohebe* Garn.-Jones species of Plantaginaceae [55]. In angiosperms, the gynoecium vasculature is generally provided by three bundles per carpel: one follows along its median plane, called dorsal or median bundle, and the other two continue along its margins, called lateral bundles [56, 57], although carpels can have one, three, five or more traces per carpel. In the studied species, the gynoecium is tricarpellary with one dorsal bundle at each carpel. In the transverse section of style, only one dorsal bundle and one pollen-tube transmitting tissue were observed (Fig 3). In the floral vasculature description of some *Polygonaceae* species, the vasculature of gynoecium comprises three or two dorsal bundles which, after their departure leave behind a ventral plexus. This plexus breaks up into three or two ventral strands and furnish an ovular trace. In some species *of Polygonum* and *Rumex* L., the ventrals do not become distinct. The ovular trace supplies the single ovule where it is completely used up. The dorsals continue in the style and terminate in the carinal stigma. The ventrals may run up to different heights in the ovary wall.

In the studied species, the ovary consisted of 3 united carpels forming one locule, so it is pseudomonomerous (Figs 3 and 4), which encloses only one orthotropous ovule with basal placentation in *P*. *equisetiforme* and *P*. *aviculare* and apical placentation in *P*. *maritimum*. The terminal part of the gynoecium is formed by 3 styles each of which ends with a stigma as in other *Polygonaceae* [28]. Three styles connate at the base as observed in our study were also found in *P*. *salicornioides* Jaub. & Spach ex Boiss and *Atraphaxis ovczinnikovii* Czukav. [29]. On the contrary, in other *Polygonaceae* such as the species of *Atraphaxis* L. sect. *tragopyrum* the three styles are free with large stigmata [58]. The difference in ovary size between LS and SS flowers was very negligible as observed in *P*. *jucundum* [31]. Since the length of the stigma is negligible, the difference in style length is the fundamental parameter to be determined when looking for the difference in stigma height between the two morphs. Polymorphism in stigma size has been shown in many distylous species. For instance, in *Linum grandiflorum* Desf. and *L*. *pubescens* Banks & Sol., the stigma of long morphs is larger, as was observed in our studied species. In contrast, in others species such as *Polygonium jucundum* [24] and *P*. *hastato-sagittatum* [30], the short morph stigma is larger than that of long morphs. The larger stigma might be able to receive more pollen grains. [33] showed that the differences in the style and stigma of LS and SS flowers of same heterostylous species of Rubiaceae (*Psychotria chiapensis* Standl. and *Psychotria poeppigiana* Müll. Arg.) is the result of structural differences developed early and maintained throughout development. In contrast, in *Guettarda scabra* L. (Rubiaceae) the differences in style heights between flowers of the two morphs were related to a growth rate reduction of the short styles development [59].

The young fruit is externally limited by a well-developed exocarp, a mesocarp formed by several layers of parenchymatous cells, and an internalendocarp (Fig 6). In most species of *Polygonaceae*, during the development, the exocarp increases in thickness and becomes sclerified. However, the mesocarp and endocarp have collapsed. On the other hand, the outer mesocarp cells layers of the young fruit, adjacent to the exocarp are smaller in size than the inner ones with the presence of several vascular bundles in this part. In correlation with what has been described in other *Polygonaceae* indicating the presence of 2 zones in the mesocarp, an outer pigmented zone with vascular tissue and an inner non-pigmented zone [60]. During the pericarp development, the most important event is the transformation of the exocarp [18]. Since the primary role of the pericarp is to protect the embryo [61], the exocarp can be considered as the most important part of the pericarp that helps seed protection. Unlike the pericarp, which becomes hard during development to ensure the protection of the seeds, the seed coat remains parenchymatous or may even be reduced or disappear during development. Indeed, the young seed coat is composed by a single layer of exotesta, single layer of mesotesta, single layer of endotesta, and a tegmen with 1–2 layers of palisade cells. In contrast, the mature seed coat is formed with fragmented layers of cells. In *P*. *maritimum*, the young seed coat has the thicker exotesta with larger thick-walled cells. The exocarp morphology in *Polygonum sec*. *Polygonum* showed the presence of two kinds of cell structure. The first type is characterized by the presence of convolute anticlinal walls with numerous folds and gaps in the walls and rectangular lumina with dendritic branches along the sides [18]. This is in accordance with our results regarding *P*. *aviculare* and *P*. *equisetiforme* (Fig 6). Similarly, [28] found that *P*. *odoratum* (Mill.) Druce has anticlinal cell walls with numerous pits set in a star-like pattern. The exocarp of *P*. *maritimum* belongs to the second cell type, which consists of narrow rectangular cells with straight anticlinal walls and broad lumen.

The achene sizes of the studied species are between 1.9–4.05 mm (length) and 1.46–2.98 mm (width). *P*. *maritimum* has the largest achenes while the smallest achene was measured in *P*. *aviculare*. Similar size to those examined in this study has been encountered in other *Polygonum* species like *P*. *swatchense* Small and *P*. *austiniae* Greene [62]. Achene surfaces are classified according to their main sculpture and additional ornaments [63]. When the exocarp increases in size during fruit maturation, thickening occurs often on the anticlinal walls of the cell and due to space constraints, the anticlinal walls become variously undulated, and such undulation on the outer surface form tubercles on the anticlinal cell walls. Our study revealed two types of achene surface, a smooth-undulate surface in the achenes of *P*. *maritimum*, and one that is striate-tubercled near the edges in *P*. *equisetiforme* and *P*. *aviculare* mature achenes. The smooth and glossy surfaces increase water repellence and decrease the risk of attack by fungi and other pathogens [15]. The functions of the verrucae are not quite clear, but similarly to some *Polygonum* species [60. 63], these thin-walled invaginations of the outer periclinal walls of exocarp cells, possibly ease absorption of water for germination and might serve for penetration of water to the inside of cells. [63] reported that the achenes are usually striate-verrucate in the xerophytic *Polygonum species* (*P*. *rurivagum*, *P*. *neglectum*, *P*. *aviculare* and some specimens of *P*. *arenastrum*).

## Conclusions

*Polygonum equisetiforme* and *P*. *aviculare* are a typically distylous species from the morphological point of view. Long and short morphs differ in stigma height and width, filament length and stamen height, and pollen grain size. *P*. *maritimum* is homostylous. The tepal vascularization is ensured by a single main vein in *P*. *maritimum* and by. one main vein and two secondary veins in the other species. In the mature anther, the epidermis and endothecium cells width were higher in *P*. *maritimum* than in *P*. *aviculare*. The detailed anatomy of the seed and achene anatomy clarified the close resemblance of these *Polygonum* species. Our investigation provides the first floral vasculature study of *P*. *equisetiforme* showing that the tepals traces usually arise independently. The eight stamens are arranged in 5+3 manner and the staminal bundle arises independently.

The results of this anatomical study showed that there is no significant difference between the two varieties of *P*. *equisetiforme*, so it seems that it is not a question of two separate varieties but a simple adaptation to the climatic conditions. Indeed, *P*. *equisetiforme* var. *peyerinhoffi* has a creeping port that makes it easier to absorb the maximum amount of moisture and to reduce the contact with the open air to minimize transpiration, which is consistent with [64] systematic classification indicating a taxonomic grouping of these two varieties. Moreover, further molecular studies on this species will help to elucidate its true systematic position within the genus. These floral anatomical descriptions, fruit development, and the vascularization constitute an original contribution, as these species had never been studied previously from those points of views.

## References

[1] Brummitt RK. Vascular plant families and genera. Royal Botanic Gardens, Kew; 1992.

[2] Heywood VH. Scanning electron microscopy in the study of plant materials. Micron. 1969; 1: 1–14.

[3] Budel JM, Farago PV, Duarte MdR, Takeda IJ. Morpho-anatomical study of the cladodes of *Homalocladium platycladum* (FJ Muell.) LH Bailey (Polygonaceae). Rev Bras Farmacogn. 2007; 17: 39–43.

[4] Koochak H, Seyyednejad SM, Motamedi H. Preliminary study on the antibacterial activity of some medicinal plants of Khuzestan (Iran). Asian Pac J Trop Med. 2010; 3: 180–184.

[5] Li A. Polygonaceae AL Jussieu. Flora of China. 2003; 5: 277–350.

[6] Brandbyge J. Polygonaceae Flowering Plants· Dicotyledons: Springer; 1993 pp. 531–544.

[7] Ronse Decraene LP, Akeroyd J. Generic limits in *Polygonum* and related genera (Polygonaceae) on the basis of floral characters. Bot J Linn Soc. 1988; 98: 321–371.

[8] Pottier-Alapetite G. Flore de la Tunisie: Angiospermes-Dicotyledones. 1. Apetales-Dialypetales. Ministère de ĺ Enseignement Supérieur et de la Recherche Scientifique et le Ministère de ĺ Agriculture; 1979.

[9] Haraldson K. Anatomy and taxonomy in Polygonaceae subfam. Polygonoideae Meisn. emend. Jaretzky. Acta Universitatis Upsaliensis, Symbolae Botanicae Upsalienses; 1978.

[10] Lersten N, Curtis J. Foliar anatomy of *Polygonum* (Polygonaceae): survey of epidermal and selected internal structures. Plant Syst Evol. 1992; 182: 71–106.

[11] Yasmin G, Khan MA, Shaheen N, Hayat MQ. Micromorphological investigation of foliar anatomy of genera *Aconogonon* and *Bistorta* of family Polygonaceae. Intl J Agric Biol. 2009; 11: 285–289.

[12] Hedberg O. Pollen morphology in the genus *Polygonum s.l.* and its taxonomic significance. Svensk Botanisk Tidskrift. 1946; 40: 371–404.

[13] Hong SP, Hedberg O. Parallel evolution of aperture numbers and arrangement in the genera *Koenigia*, *Persicaria* and *Aconogonon* (Polygonaceae). Grana. 1990; 29: 177–184.

[14] Nowicke JW, Skvarla JJ. Pollen morphology and the relationship of the Plumbaginaceae, Polygonaceae, and Primulaceae to the order Centrospermae. Smithsonian Contributions to Botany. 1977; 37: 1–64.

[15] Barthlott W. Epidermal and seed surface characters of plants: systematic applicability and some evolutionary aspects. Nord J Bot. 1981; 1: 345–355.

[16] Martin AC. Identifying polygonum seeds. The Journal of Wildlife Management. 1954; 18: 514–520.

[17] Yurtseva O, Yakovleva N, Ivanova Radkevich T. Heterocarpy in *Polygonum aviculare* L. *s. str.* and related species (*Polygonum*, subsect. *Polygonum*). Bulletin of Moscow Society of Naturalists. 1999; 104: 13–20.

[18] Ronse Decraene LP, Hong SP, Smets E. Systematic significance of fruit morphology and anatomy in tribes Persicarieae and Polygoneae (Polygonaceae). Bot J Linn Soc. 2000; 134: 301–337.

[19] Bauer R. Entwicklungsgeschichtliche untersuchungen an Polygonaceenbluten. Flora. 1922; 115: 272–292.

[20] Emberger L. 1939. Aperçu général sur la végétation du Maroc: commentaire de la carte phytogéographique du Maroc 1: 1 500 000. H. Huber Berne; 1939.

[21] Ronse Decraene LP, Smets E. The floral development of *Popowia whitei* (Annonaceae). Nord J Bot. 1990; 10: 411–420.

[22] Hildebrand F. Die geschlechter-vertheilung bei den pflanzen. Engelmann, Leipzig; 1867.

[23] Hassan M, Khan M. Style-stamen dimorphism in *Polygonum* L. (Polygonaceae). Bangl Jf Bot. 1987; 16: 93–95.

[24] Reddy NP, Bahadur B, Kumar PV. Heterostyly in *Polygonum chinense* L. J Genet. 1977; 63: 79.

[25] Chen ML, Zhang XP. Distyly in *Polygonum jucundum* Meisn.(Polygonaceae). Plant Syst Evol. 2010; 288: 139–148.

[26] Lloyd D, Webb C. The evolution of heterostyly. Evolution and function of heterostyly: Springer; 1992 pp. 151–178.

[27] Hong SP. The dimorphic heterostyly in *Aconogonon campanulatum* (Polygonaceae). Plant Syst Evol. 1991; 176: 125–131.

[28] Kantachot C, Chantaranothai P. Achene morphology of *Polygonum sl* (Polygonaceae) in Thailand. Trop Nat History. 2011; 11: 21–28.

[29] Chen ML. Floral morphology and breeding system in *Polygonum hastato-sagittatum* Mak.(Polygonaceae). Flora. 2012; 207: 365–371.

[30] Huang LJ, Fu WL, Wang XF. Floral development at multiple spatial scales in *Polygonum jucundum* (Polygonaceae), a distylous species with broadly open flowers. PloS one. 2014; 9: e102802 10.1371/journal.pone.0102802 25058669 PMC4109959

[31] Wang CH, Du W, Wang XF. Reproductive investment in a cleistogamous morph of *Polygonum jucundum* (Polygonaceae). Plant Syst Evol. 2017; 303: 559–563.

[32] Schneider CA, Rasband WS, Eliceiri KW. NIH Image to ImageJ: 25 years of image analysis. Nat Methods. 2012; 9: 671–675. 10.1038/nmeth.2089 22930834 PMC5554542

[33] Faivre AE. Ontogenetic differences in heterostylous plants and implications for development from a herkogamous ancestor. Evolution. 2000; 54: 847–858. 10.1111/j.0014-3820.2000.tb00085.x 10937258

[34] Sampson BJ, Stringer SJ, Marshall DA. Blueberry floral attributes and their effect on the pollination efficiency of an oligolectic bee, *Osmia ribifloris* Cockerell (Megachilidae: Apoidea). HortScience. 2013; 48: 136–142.

[35] De Castro CC, Araujo AC. Distyly and sequential pollinators of *Psychotria nuda* (Rubiaceae) in the Atlantic rain forest, Brazil. Plant Syst Evol. 2004; 244: 131–139.

[36] Koch AK, Silva P, Silva CA. Biologia reprodutiva de *Psychotria carthagenensis* (Rubiaceae), espécie distílica de fragmento florestal de mata ciliar, Centro-Oeste do Brasil. Rodriguésia-Instituto de Pesquisas Jardim Botânico do Rio de Janeiro. 2010; 61: 551–558.

[37] Deng Y, Chen S, Teng N, Chen F, Li F, Song A, Guan Z. Flower morphologic anatomy and embryological characteristics in Chrysanthemum multicaule (Asteraceae). Sci Hortic. 2010; 124: 500–505.

[38] Rao BH, Rao PP. Sporogenesis and gametogenesis of some Boraginaceae. Feddes Repertorium. 1992; 103: 35–40.

[39] Zulkarnain Z. Embryology of *Swainsona formosa* (Fabaceae): anther and ovule development. J Bioscience. 2005; 12: 11.

[40] Chehregani A, Tanomi N. Ovule ontogenesis and megagametophyte development in Onobrychis schahuensis Bornm.(Fabaceae). Turk J Bot. 2010; 34: 241–248.

[41] Zou F, Sheng-Lin C, De-Yi Y, Zhang RQ, Zhang L, Xiong H. Microsporogenesis, megasporogensis and male and female gametophyte development in Feijoa sellowiana (Myrtaceae). Int J Agric Biol. 2016; 18.

[42] Zhang Q, Hao Q, Guo X, Liu Q, Sun Y, Liu Q, Wang K. Anther and ovule development in Camellia japonica (Naidong) in relation to winter dormancy: Climatic evolution considerations. Flora. 2017; 233: 127–139.

[43] Yang S, Peng H, Liang H. Embryological observation on *Camellia yunnanensis* var. *camellioides* with a comparison of embryological features in *Camellia*. Guihaia, 2002; 22:340–344.

[44] Ghimire B, Suh GU, Lee CH, Heo K, Jeong MJ. Embryological studies on *Abelia tyaihyoni* Nakai (Caprifoliaceae). Flora. 2018; 242: 79–88.

[45] Hong SP, Oh IC, De Craene LR. Pollen morphology of the genera *Polygonum s. str*. and *Polygonella* (Polygoneae: Polygonaceae). Plant Syst Evol. 2005; 254: 13–30.

[46] Yurtseva O, Severova E, Bovina IY. Pollen morphology and taxonomy of Atraphaxis (Polygoneae, Polygonaceae). Plant Syst Evol. 2014; 300: 749–766.

[47] Zhang X, Zhou Z. A study on pollen morphology and its phylogeny of'Polygonaceae in China. China Science and Technology University Press; 1998.

[48] Yasmin G, Khan MA, Shaheen N, Hayat MQ. Taxonomic significance of leaf epidermal anatomy of selected *Persicaria* Mill. species of family Polygonaceae from Pakistan. Afric J Biotechnol. 2010; 9: 3759–3768.

[49] Nuraliev MS, Sokoloff DD, Oskolski AA. Floral anatomy of Asian *Schefflera* (Araliaceae, Apiales): Comparing variation of flower groundplan and vascular patterns. Int J Plant Sci. 2011; 172: 735–762.

[50] Von Balthazar M, Endress PK. Development of inflorescences and flowers in Buxaceae and the problem of perianth interpretation. Int J Plant Sci. 2002; 163: 847–876.

[51] Laubengayer RA. Studies in the anatomy and morphology of the polygonaceous flower. Am J Bot. 1937; 24: 329–343.

[52] Tucker SC. Floral ontogeny in Sophoreae (Leguminosae: Papilionoideae). III. Radial symmetry and random petal aestivation in Cadia purpurea. Am J Bot. 2002; 89: 748–757. 10.3732/ajb.89.5.748 21665674

[53] Tucker SC. Overlapping organ initiation and common primordia in flowers of Pisum sativum (Leguminosae: Papilionoideae). Am J Bot. 1989; 76: 714–729

[54] Sundberg MD. Short communication petal‐stamen initiation in the genus Cyclamen (Primulaceae). Am J Bot. 1982; 69: 1707–1709.

[55] Bello MA, Rudall P, González F, Fernández-Alonso JL. Floral morphology and development in *Aragoa* (Plantaginaceae) and related members of the order Lamiales. Int J Plant Sci. 2004; 165: 723–738.

[56] Endress PK. Floral structure and evolution of primitive angiosperms: recent advances. Plant Syst Evol. 1994; 192: 79–97.

[57] Lord JM. Flower and fruit. Morphology, ontogeny, phylogeny, function, ecology. New Zeal J Bot. 2011; 49: 141–142.

[58] Neubauer BF. The development of the achene of *Polygonum pensylvanicum*: embryo, endosperm, and pericarp. Am J Bot. 1971; 58: 655–664.

[59] Richards J, Koptur S. Floral variation and distyly in *Guettarda scabra* (Rubiaceae). Am J Bot. 1993; 80: 31–40.

[60] Yurtseva O, Kuznetsova O, Mavrodieva M, Mavrodiev E. What is Atraphaxis L.(Polygonaceae, Polygoneae): cryptic taxa and resolved taxonomic complexity instead of the formal lumping and the lack of morphological synapomorphies. PeerJ. 2016; 4: e1977 10.7717/peerj.1977 27168986 PMC4860328

[61] Roth I. Fruits of angiosperms. Encyclopedia of plant anatomy. 1977.

[62] Costea M, Tardif FJ. The biology of Canadian weeds. 131. *Polygonum aviculare* L. Can J Plant Sci. 2005; 85: 481–506.

[63] Yurtseva O. Ultrasculpture of achene surface in *Polygonum* section *Polygonum* (Polygonaceae) in Russia. Nordic J Bot. 2001; 21: 513–528.

[64] Le Floch É, Boulos L, Vela E. Catalogue synonymique commenté de la flore de Tunisie. Simpact; 2010.

